# Mono- and Bimetallic Nanoparticles Stabilized by an Aromatic Polymeric Network for a Suzuki Cross-Coupling Reaction

**DOI:** 10.3390/nano12010094

**Published:** 2021-12-29

**Authors:** Linda Zh. Nikoshvili, Kristina N. Shkerina, Alexey V. Bykov, Alexander I. Sidorov, Alexander L. Vasiliev, Mikhail G. Sulman, Lioubov Kiwi-Minsker

**Affiliations:** 1 Department of Biotechnology, Chemistry and Standardization, Tver State Technical University, Afanasy Nikitina Street 22, 170026 Tver, Russia; kshkerina@mail.ru (K.N.S.); bykovav@yandex.ru (A.V.B.); sidorov@science.tver.ru (A.I.S.); sulmanmikhail@yandex.ru (M.G.S.); 2 National Research Centre “Kurchatov Institute”, Kurchatov Square 1, 123182 Moscow, Russia; a.vasiliev56@gmail.com; 3 Institute of Crystallography of the Russian Academy of Sciences, Leninsky Prospekt 59, 117333 Moscow, Russia; 4 Regional Technological Centre, Tver State University, Zhelyabova Street 33, 170100 Tver, Russia; 5 Department of Basic Sciences, Ecole Polytechnique Fédérale de Lausanne, ISIC-FSB-EPFL, CH-1015 Lausanne, Switzerland

**Keywords:** Suzuki cross-coupling, hyper-cross-linked polystyrene, palladium, nanoparticles, bimetallic catalysts

## Abstract

This work addresses the Suzuki cross-coupling between 4-bromoanisole (BrAn) and phenylboronic acid (PBA) in an environmentally benign ethanol–water solvent catalysed by mono- (Pd) and bimetallic (PdAu, PdCu, PdZn) nanoparticles (NPs) stabilised within hyper-cross-linked polystyrene (HPS) bearing tertiary amino groups. Small Pd NPs of about 2 nm in diameters were formed and stabilized by HPS independently in the presence of other metals. High catalytic activity and complete conversion of BrAn was attained at low Pd loading. Introduction of Zn to the catalyst composition resulted in the formation of Pd/Zn/ZnO NPs, which demonstrated nearly double activity as compared to Pd/HPS. Bimetallic core-shell PdAu/HPS samples were 3-fold more active as compared to Pd/HPS. Both Pd/HPS and PdAu/HPS samples revealed promising stability confirmed by catalyst recycling in repeated reaction runs.

## 1. Introduction

Over the last decades, many efforts have been devoted to the development of heterogeneous catalysts based on nanoparticles (NPs) of noble metals due to their unique catalytic properties [1]. However, the high cost of noble metals still significantly limits their use in industrial catalysis. At the same time, metal NPs tend to agglomerate and are easily oxidized in air, resulting in a loss of their catalytic activity. Thus, the development of cost-effective materials with limited aggregation and leaching of noble metal NPs is of high importance [2]. In recent years, porous organic polymers with a high surface area have attracted great attention. They are widely used for adsorption and gases separation, energy storage, as well as in heterogeneous catalysis [3,4,5,6].

Hyper-cross-linked polymers are promising supports for heterogeneous catalysts due to their low cost, high chemical stability in various media, high surface area and pore volume, and availability of a variety of monomers, allowing adjustable pore sizes and surface chemistry [7]. A high degree of cross-linking prevents the destruction of polymer chains and their collapse into a dense non-porous material, making them attractive catalysts for fine organic synthesis [8].

Pd is known to effectively catalyze cross-coupling reactions, which are ones of the most widely used processes in fine organic synthesis. The most promising strategy for reducing the environmental impact during this process is to use catalysts with a low Pd loading, which would be easy in preparation, highly active/selective, and recyclable [9]. It should be emphasized that Pd-catalyzed coupling reactions are complicated by various processes (Pd aggregation, precipitation, leaching, etc.) and contribute to the changes in catalyst properties during the reaction [10,11], since in the course of cross-coupling palladium constantly changes its oxidation state from Pd^0^ to Pd^2+^ and vice versa while passing trough oxidative addition of aryl halide, transmetalation with arylboronic acid, and reductive elimination of the cross-coupling product [12]. Thus, the reaction mechanism always includes homogeneous stages.

Besides monometallic Pd-based catalysts, bimetallic systems are also used in Suzuki cross-coupling. Among them, Pd-Au is the most promising since Pd and Au can form core-shell NPs, which exhibit higher catalytic activity, selectivity, and stability as compared to monometallic analogues [13,14,15,16,17]. Bimetallic Pd-Cu [18] and Pd-Zn catalysts have not been well studied in Suzuki cross-coupling. Cu and Zn compounds are mainly used as additives to the reaction mixture, while Pd catalyzes cross-coupling process. For example, it was shown [19,20] that copper compounds (CuCl > CuBr, Cu_2_O > CuI) are effective dopants of Suzuki cross-coupling catalyzed by palladium acetate. It was proposed that CuCl in the reaction mixture accelerates the transmetalation step. Zinc dihalides were also reported as the transmetalating accelerators in Suzuki cross-coupling [21]. While the NPs of ZnPd alloys were reported to boost the activity/selectivity of various Pd-catalyzed processes [22,23,24,25,26], to the best of our knowledge, they were never tested in Suzuki cross-coupling.

**Scheme 1:**
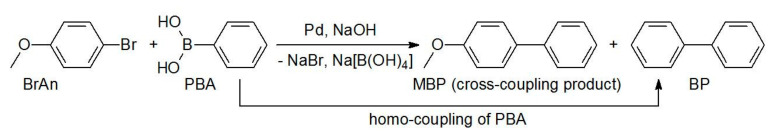
Simplified representation of Suzuki cross-coupling between BrAn and PBA.

In this work we have synthesized a series of mono- (Pd) and bimetallic (PdAu, PdCu, PdZn) NPs using hyper-cross-linked polystyrene (HPS) as catalytic support. For the first time we have shown that the formation of PdZn alloy NPs is beneficial for Suzuki cross-coupling between 4-bromoanisole (BrAn) and phenylboronic acid (PBA) as model compounds (see Scheme 1). The choice of these model compounds was due to the formation of 4-methoxybiphenyl (MBP) as a target cross-coupling product, which can be easily detected separately of the side product biphenyl (BP)—a result of PBA homo-coupling. Moreover, aryl bromides have moderate reactivity in comparison with aryl iodides, which makes them convenient substrates for kinetic study and comparable evaluation of catalytic activity. It was also shown that PdAu/HPS catalysts are the most active among the synthesized samples allowing a lower amount of boronic acid and base.

Earlier we have shown that Au(core)-Pd(shell) NPs are formed in the pores of HPS during the catalyst calcination in gaseous hydrogen while using HAuCl_4_ and PdCl_2_(CH_3_CN)_4_ as precursors [17]. Synthesized PdAu NPs were also found to be more active and stable as compared to the corresponding monometallic Pd/HPS sample in Suzuki cross-coupling. However, in all the preliminarily activated catalysts, an incomplete reduction of palladium as well as the presence of chlorine ions were detected, complicating the interpretation of the catalytic results. In order to avoid these issues, in this study we used palladium acetate as a precursor, which allowed a complete palladium reduction and the absence of any chlorine.

## 2. Materials and Methods

### 2.1. Materials

The hyper-cross-linked polystyrene (HPS), containing tertiary amino groups (MN100, specific surface area calculated by the Brunauer–Emmett–Teller method, BET SSA 793 m^2^/g), Purolite Int., Llantrisant, UK), was washed with distilled water, acetone, and then dried under vacuum, as described elsewhere [27]. Such a treatment eliminated iron and chlorine ions, which remained in commercial HPS after its synthesis, and also decreased the polymer wetness that was used as catalyst support.

4-Bromoanisole (BrAn, ≥98%) was purchased from Merck KGaA, Darmstadt, Germany. 4-Methoxybiphenyl (MBP, >99%) was obtained from Tokyo Chemical Industry Co. Ltd., Tokyo, Japan. Phenylboronic acid (PBA, 95%), diphenylamine (99%), biphenyl (BP, 99.5%), tetrahydrofuran (THF, ≥99.9%), ethanol (EtOH, ≥99.8%), isopropanol (*i*-PrOH, ≥99.5%), chloroform (CHCl_3_, ≥99%, anhydrous, containing 0.5% EtOH as a stabilizer), sodium carbonate (Na_2_CO_3_, ≥99.5%), sodium hydroxide (NaOH, ≥98%), copper acetate monohydrate (Cu(CH_3_COO)_2_·H_2_O, ≥98%), and zinc acetate dihydrate (Zn(CH_3_COO)_2_·2H_2_O, ≥99%) were purchased from Sigma-Aldrich, St. Louis, MO, USA. Palladium acetate (Pd(CH_3_COO)_2_, 47.68%ofPd) and gold(III) chloride hydrate (HAuCl_4_·nH_2_O, 48.95% of Au) were obtained from JSC “Aurat”, Moscow, Russia. All chemicals were used as received. Distilled water was purified with an Elsi-Aqua water purification system.

### 2.2. Catalyst Synthesis and Characterization

HPS-based catalysts containing Pd were synthesized via the wet-impregnation method. In a typical experiment, 1 g of pretreated, dried, and crushed (<63 μm) granules of HPS were impregnated with 2.8 mL of the THF solution of precursor (Pd(CH_3_COO)_2_ or HAuCl_4_) of a chosen concentration. Then the sample was air-dried at 70 °C, treated with 2.7 mL of Na_2_CO_3_ aqueous solution (concentration 0.07 mol/L), and dried until a constant weight was achieved. After that, the resulting samples were washed with distilled water till neutral pH and air-dried again at 70 °C until a constant weight. Thus, the following catalysts were synthesized and designated: 1%-Pd/HPS containing 1.0 wt.% of Pd (confirmed by the XFA) and 2%-Au/HPS containing 1.9 wt.% of Au. These catalysts were reduced in hydrogen flow (100 mL/min) at 300 °C for 3 h and were designated as 1%-Pd/HPS-R and 2%-Au/HPS-R.

Bimetallic PdAu/HPS catalysts were synthesized by the impregnation of reduced 2%-Au/HPS-R with the Pd precursor according to the above procedure used for a Pd-monometallic sample. Thus, two samples PdAu/HPS were synthesized: 0.5%-PdAu/HPS (containing 0.6 wt.% of Pd and 2.0 wt.% of Au) and 1%-PdAu/HPS (containing 0.9 wt.% of Pd and 2.0 wt.% of Au).

Bimetallic PdCu/HPS and PdZn/HPS catalysts were synthesized by the impregnation of HPS with Pd(CH_3_COO)_2_ and then (after drying at 70 °C) with Cu(CH_3_COO)_2_ or Zn(CH_3_COO)_2_ following the treatment by Na_2_CO_3_ solution, washing, and drying (see above). Thus, the samples 1%-PdCu/HPS (containing 1.3 wt.% of Pd and 2.0 wt.% of Cu) and 1%-PdZn/HPS (containing 1.5 wt.% of Pd and 1.9 wt.% of Zn) were synthesized.

All bimetallic samples were also reduced in a hydrogen flow at 300 °C and designated as 0.5%-PdAu/HPS-R, 1%-PdAu/HPS-R, 1%-PdCu/HPS-R, and 1%-PdZn/HPS-R. It is noteworthy that reduction temperature was limited by thermal stability of HPS, which is up to 300 °C in an argon medium (intensive destruction of the polymer starts after attaining >400 °C).

Note that all the synthesized catalyst samples (both the initial and reduced ones) were stored in air.

The catalysts were characterized by liquid nitrogen physisorption, X-ray photoelectron spectroscopy (XPS), and scanning transmission electron microscopy (STEM).

Liquid nitrogen physisorption was carried out using a Beckman Coulter SA 3100 (Coulter Corporation, Miami, FL, USA). Prior to the analysis, each sample was placed in a quartz cell installed in the Becman Coulter SA-PREP. The samples were pretreated over 60 min under nitrogen at 120 °C. Once the pretreatment was completed, the cell was cooled and weighed, and then transferred to the analytical port. Analysis was performed at −196 °C and at relative pressure of 0.9814 (for pores less than 100 nm in diameter) to obtain a PSD (ADS) profile.

XPS data were obtained using Mg Kα (hν = 1253.6 eV) radiation with an ES-2403 spectrometer (Institute for Analytic Instrumentation of RAS, Saint Petersburg, Russia) equipped with an energy analyzer PHOIBOS 100-MCD5 (SPECS, Berlin, Germany) and X-ray source XR-50 (SPECS, Berlin, Germany). All the data were acquired at X-ray power of 250 W. Survey spectra were recorded at an energy step of 0.5 eV with an analyzer pass energy of 40 eV. High-resolution spectra were recorded at an energy step of 0.05 eV with an analyzer pass energy of 7 eV. Samples were outgassed for 180 min before analysis and were stable during the examination. The data analysis was performed via Casa XPS. Binding energies (BEs) were determined with the error ±0.1 eV.

STEM characterization was carried out using an FEI Tecnai Osiris instrument (Thermo Fisher Scientific, Waltham, MA, USA) operating at an accelerating voltage of 200 kV, equipped with a high-angle annular dark field (HAADF) detector (Fischione, Export, PA, USA) and an energy-dispersive X-ray (EDX) microanalysis spectrometer (EDAX, Mahwah, NJ, USA). Samples were prepared by embedding them in epoxy resin followed by microtoming (ca. 50 nm thick) at ambient conditions. For the image processing, Digital Micrograph (Gatan, Pleasanton, CA, USA) software and TIA (Thermo Fisher Scientific, Waltham, MA, USA) were used. A Holey carbon/Cu grid was used as a sample support.

### 2.3. Reaction Procedure and Analysis of the Reaction Mixture

The Suzuki cross-coupling was carried in a temperature-controlled glass batch reactor with a magnetic stirrer, with variation in the following parameters.

The following series of experiments were carried out:

(i) Stirring rate from 500 rpm to 1300 rpm using 1 mmol of BrAn, 1.5 mmol of PBA, 1.5 mmol of NaOH, and 30 mg of catalyst (corresponds to 0.28 mol.% of Pd with respect to BrAn) at 60 °C;
(ii) Catalyst loading in the range from 15 mg (0.14 mol.% of Pd) up to 50 mg (0.47 mol.% of Pd) using 1 mmol of BrAn, 1.5 mmol of PBA, and 1.5 mmol of NaOH at 60 °C and 900 rpm;
(iii) Amounts of PBA (1.5–3.0 mmol) and NaOH (1.5–3.8 mmol) using 1 mmol of BrAn and 30 mg of catalyst at 60 °C and 900 rpm;
(iv) Reaction temperature (40–70 °C) using 1 mmol of BrAn, 2.5 mmol of PBA, 3.0 mmol of NaOH, and 30 mg of catalyst at 900 rpm.

All experiments were carried out in air using an EtOH-H_2_O mixture (volumetric ratio 4:1) as a solvent. The total volume of the liquid phase was 50 mL. The choice of EtOH-H_2_O mixture was first, due to the environmentally benign nature of these solvents, and second, to ensure that the system remained homogeneous in the range of the selected concentrations of the reactants (BrAn, PBA, and NaOH) and the products (MBP and BP). It is worthy to mention that the existing protocols of Suzuki cross-coupling allow using a wide range of bases, which mainly are sodium and potassium carbonates. The choice of NaOH as a base was due to our preliminarily studies [28], which revealed that sodium hydroxide provides the highest initial reaction rates possibly due to its better solubility in the chosen solvents. Before the catalyst addition in the reactor, in each experiment the blank test (duration of 60 min) was carried out in order to ensure that there is no reaction without the catalyst. The necessity of a blank test was due to the fact that even small amounts of leached palladium, if remaining on the stir bar or on the reactor walls, can catalyze the cross-coupling [29]; thus, there is a possibility that the reaction will proceed in the absence of a catalyst. In all experiments, PBA was used in excess with respect to BrAn due to the possible non-selective PBA homo-coupling with the formation of BP.

In each catalytic experiment, samples of the reaction mixture were periodically taken and analyzed via GC-MS (Shimadzu GCMS-QP2010S) equipped with a capillary column HP-1MS (100 m × 0.25 mm i.d., 0.25 μm film thickness). Helium was used as a carrier gas at a pressure of 74.8 kPa and linear velocity of 36.3 cm/s. Oven temperature was programmed: 120 °C (0 min) → 10 °C/min (160°C) → 25 °C/min (300°C) → 300 °C (2.4 min). The temperature of the injector, interface, and ion source was at 260 °C, ranging from 10 up to 500 *m*/*z*. The concentrations of the reaction mixture components were calculated using the internal standard calibration method (diphenylamine was used as an internal standard). It is noteworthy that during the GS-MS analysis, PBA underwent dehydration and trimerization, which affected the signal intensity. Due to the low level of confidence in the quantitative analysis of PBA, its consumption was not monitored.

Catalytic activity was characterized by the initial transformation rate, *R*_0_, defined as the tangent of the slope of the initial linear part on the kinetic curves of BrAn consumption, and related to the amount of palladium in the system: *R*_0_ = (*N_BrAn_*_,0_ − *N_BrAn,i_*) × *τ_i_*^−1^ × *N_Pd_*^−1^, where *N_BrAn_* is a number of moles of BrAn transformed by the reaction time *τ*; *N_Pd_* is the overall number of moles of Pd; and *τ* is the reaction time, in minutes.

The conversion (*X*,%) of BrAn was defined as *X*,% = (*N_BrAn_*_,0_ − *N_BrAn,i_*) × *N_BrAn_*_,0_^−1^ × 100.

### 2.4. Catalysts Separation from the Reaction Mixture for Reuse

After the reaction completion, the catalysts were filtered under vacuum using a membrane filter (Nylon, 0.45 μm pore size, 50 mm diameter) and sequentially washed with EtOH (30 mL), i-PrOH (30 mL), water (500 mL), i-PrOH (30 mL), and chloroform (50 mL). Then they were dried till constant weight at 70 °C. It is noteworthy that for the repeated use, several (at least 2) catalyst samples were collected from the previous reaction runs and the averaged catalyst sample was taken for the next run. In this way, all the reaction conditions remained unchanged, including the catalyst weight.

### 2.5. Hot-Filtration Test

To ensure that there is no homogeneous palladium active species, which can catalyze the cross-coupling in the absence of HPS-based catalysts, hot filtration was carried out. After 1 min, 10 mL of the reaction mixture was immediately separated using a syringe equipped with a PTFE membrane (0.22 µm pore size) and transferred to the preliminarily thermostated second glass batch reactor. After that, the reaction was carried out under the same conditions for 60 min, with periodic sampling and analysis.

## 3. Results

### 3.1. Catalyst Characterization

**Figure 1:**
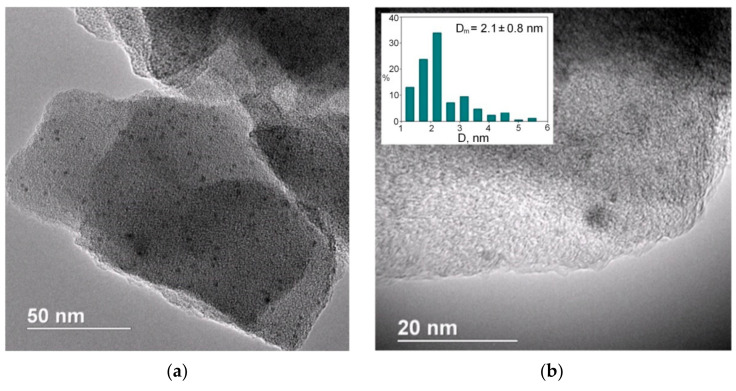
Bright-field STEM images of the reduced sample 1%-Pd/HPS-R at different magnification: scale 50 nm (**a**) and scale 20 nm (**b**).

The liquid nitrogen physisorption revealed that the initial HPS and all the fresh, unreduced catalysts have a predominant microporosity (see Table 1 and Figure S1a,c). The introduction of palladium and gold into the HPS led to the decrease in both the SSA of the micropores and the external SSA. The subsequent reduction in hydrogen flow (see, e.g., 1%-Pd/HPS-R and 0.5%-PdAu/HPS-R) led to a further decrease in SSA, caused by blockage of the micro-, meso- and macropores along with the formation of NPs. According to the data of STEM, in the case of the monometallic sample (1%-Pd/HPS-R), Pd NPs with a mean diameter of 2.1 ± 0.8 nm were formed after the reduction (Figure 1).

**Figure 2:**
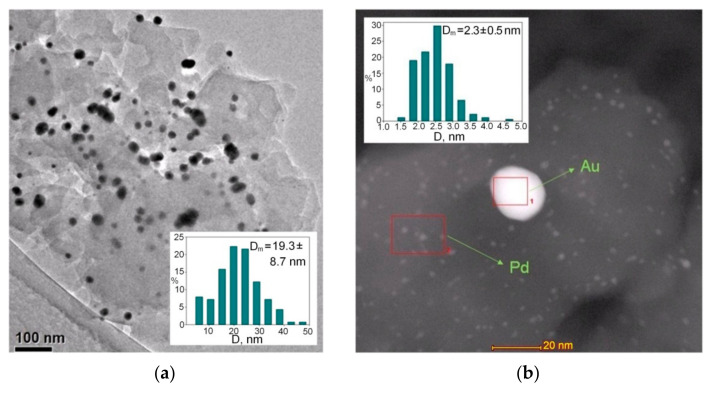
Bright-field (**a**) and dark-field (**b**) (scale 20 nm) STEM images of the reduced sample 0.5%-PdAu/HPS-R.

In the sample 0.5%-PdAu/HPS-R, the Au NPs with a mean diameter of 19.3 ± 8.7 nm as well as Pd NPs with a mean diameter of 2.3 ± 0.5 nm were found. Earlier, for the 0.4%-PdAu/HPS-R catalyst (synthesized using PdCl_2_(CH_3_CN)_2_ as a precursor), we have shown that Pd atoms form a very thin layer (2–3 atomic monolayers) on the surface of the preliminarily created Au NPs [17]. From the dark-field STEM image of the 0.5%-PdAu/HPS-R sample used in this work (Figure 2b), it can be seen the separately located small Pd NPs and big NPs, which can be ascribed to Au, since, to the best of our knowledge, palladium acetate never forms NPs of about 20 nm in diameter in the HPS environment at a metal content of 0.5–1 wt.%. At the same time, EDX data (Figure S2) have shown a weak signal of Pd in the area 1 of Au NPs, which suggests a core-shell structure.

For the unreduced monometallic sample, 1%-Pd/HPS, the XPS analysis revealed the following chemical states of Pd 3d_5/2_: Pd(CH_3_COO)_2_ (BE is 338.4 eV), PdO (BE is 337.1 eV), and a partially oxidized surface of metallic palladium PdO/Pd(BE is 336.5 eV) [30]. It is noteworthy that the value of BE of 336.5 eV also corresponds to small Pd_n_ clusters [31]. After the reduction (1%-Pd/HPS-R) in a hydrogen flow, Pd^0^ NPs (BE is 335.0 eV) were found to prevail on the catalyst surface.

In the case of 0.5%-PdAu/HPS and 1%-PdAu/HPS the values of the Pd 3d_5/2_ BEs were in the range of 337.4–337.5eV (Figure S3b), which correspond to PdO [30]. At the same time, the value of BE of Au 4f_7/2_ was 85.6 eV (see e.g., Figure S3a), which indicates the presence of Au_2_O_3_ on the surfaces [30]. After the reduction (samples 0.5%-PdAu/HPS-R and 1%-PdAu/HPS-R) the value of BE of Pd 3d_5/2_ was found to be 336.5 eV (Figure S4b), which likely corresponds to the partially oxidized surface of metallic palladium PdO/Pd [30]. The BE of Au 4f_7/2_ was equal to 85.2 eV (Figure S4a). It is noteworthy that there is no reference data for this BE value of Au 4f_7/2_, but similar BEs can be found in gold alloys with other metals [30]. Thus, it can be assumed that the shift in the BE of gold is associated with the formation of a surface PdAu alloy.

**Figure 3:**
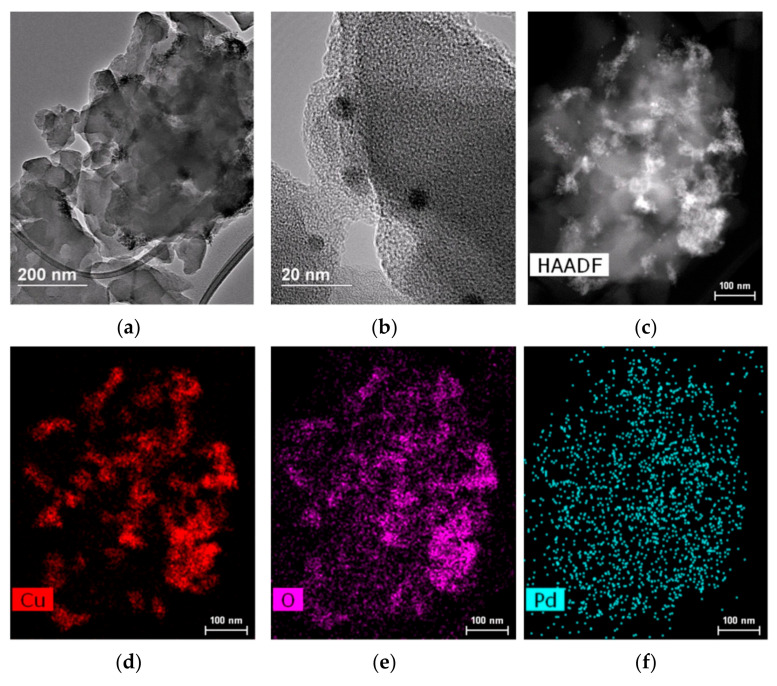
Bright-field (**a**,**b**) and dark-field (**c**) STEM images of the initial (unreduced) sample 1%-PdCu/HPS and EDX mapping of Cu (**d**), O (**e**), and Pd (**f**).

**Figure 4:**
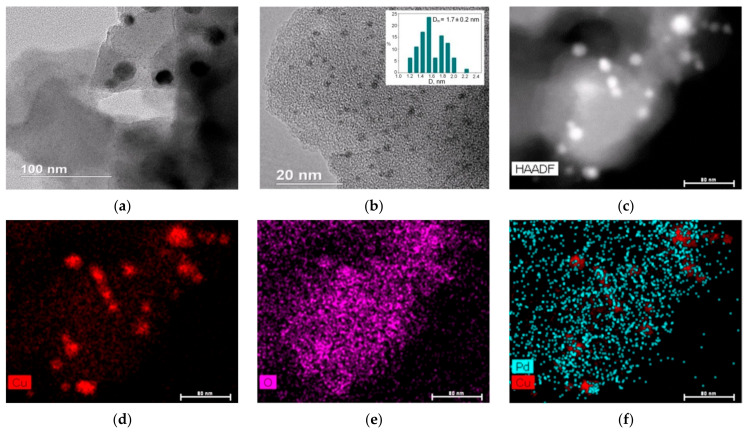
Bright-field (**a**,**b**) and dark-field (**c**) (scale 80 nm) STEM images of the reduced sample 1%-PdCu/HPS-R and EDX mapping of the Cu (**d**), O (**e**), and Cu-Pd superposition (**f**) (scales 80 nm).

In the initial (unreduced) 1%-PdCu/HPS and 1%-PdZn/HPS catalysts, a noticeable increase in SSA was observed (from 793 m^2^/g up to 872 m^2^/g and 851 m^2^/g, respectively) as compared to the initial HPS due to the increase in microporosity (Table 1, Figure S1a). As seen from Figure 3, in the 1%-PdCu/HPS sample a large amount of Cu-containing aggregates occurred (Figure 3a–e), which can artificially create a microporous surface formed by the gaps between the contacting particles, while Pd remained uniformly distributed (Figure 3f). After the reduction in a hydrogen flow (1%-PdCu/HPS-R sample) the SSA decreased by more than two times (from 872 m^2^/g to 414 m^2^/g) due to the decrease in microporosity (see Table 1 and also Figure S1b,d), which can be due to the nucleation of the Cu- and Pd-containing particles (mean diameter of Pd NPs was 1.7 ± 0.2 nm) in the polymeric environment (Figure 4a–d). Moreover, the superposition of the Cu and Pd EDX maps (Figure 4f) clearly shows that the two metals were distributed independently of each other.

In the case of 1%-PdCu/HPS (Figure S3c,d), the value of BE of Pd 3d_5/2_ was 338.2 eV (corresponds to Pd(CH_3_COO)_2_), while BE of Cu 2p_3/2_ was 936.4 eV, which likely corresponds to copper acetate [32]. For the reduced sample 1%-PdCu/HPS-R (Figure S4c,d), palladium was found in the form of partially oxidized NPs (BE of Pd 3d_5/2_ was 336.3 eV), while copper was in the form of Cu(OH)_2_ (BE of Cu 2p_3/2_ was 934.6 eV) [30].

**Figure 5:**
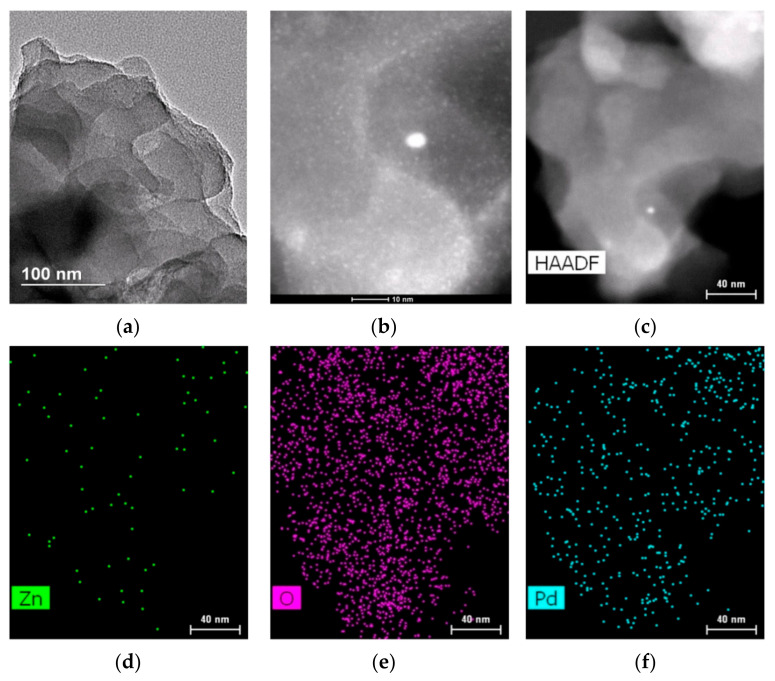
Bright-field (**a**) and dark-field (**b**,**c**) (scale is 10 nm for (**b**) and 40 nm for (**c**)) STEM images of the initial (unreduced) sample 1%-PdZn/HPS and EDX mapping of Zn (**d**), O (**e**), and Pd (**f**) (scales 40 nm).

**Figure 6:**
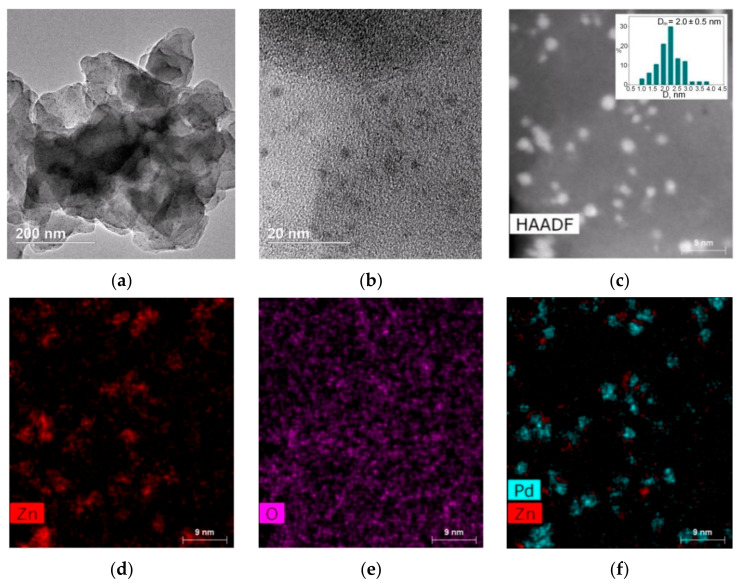
Bright-field (**a**,**b**) and dark-field (**c**)(scale 9 nm) STEM images of the reduced sample 1%-PdZn/HPS-R and EDX mapping of the Zn (**d**), O (**e**), and Zn-Pd superposition (**f**) (scales 9 nm).

In the case of 1%-PdZn/HPS, in contrast to 1%-PdCu/HPS, both zinc acetate and palladium acetate were uniformly distributed in the HPS (Figure 5). Uniform deposition of Pd and Zn compounds resulted in formation of mixed PdZn NPs with a mean diameter of 2.0 ± 0.5 nm (Figure 6) after the reduction in H_2_ flow. It is noteworthy that, according to the XPS data, palladium on the surface of unreduced 1%-PdZn/HPS sample (Figure S3e,f) was in the form of its acetate (BE of Pd 3d_5/2_ was 338.1 eV) while zinc was in the form of partially decomposed Zn(CH_3_COO)_2_ (BE of Zn 2p_3/2_ was 1023.9 eV) [33].

During the reduction treatment, palladium acetate was presumably transformed to the metallic form. The BE of Pd 3d_5/2_ on the surface of 1%-PdZn/HPS-R was 335.7 eV, which has a positive shift as compared to bulk Pd^0^ (335.0 eV), and can be explained by the donation of electron density from palladium to zinc contacting Pd directly. The BE of Zn 2p_3/2_ on the surface of 1%-PdZn/HPS-R was 1022.4 eV, which corresponds to ZnO [30].

The shift in the Pd BE has been described by many authors [22,25,26] and was mainly considered as evidence of the formation of the PdZn alloy. The corresponding shift in the BE of Zn 2p is difficult to find by the XPS method since this band of Zn has low sensitivity towards reduction changes in ZnO [25]. At the same time, zinc can be easier oxidized than palladium, which can result in the formation of a thin ZnO layer on the surface of PdZn alloy (note that all catalysts were stored in air). Thus, the obtained results suggest a partial reduction in Zn and that the synthesized bimetallic NPs had a mixed composition in the form of Pd/Zn/ZnO NPs.

### 3.2. Suzuki Cross-Coupling

Using monometallic 1%-Pd/HPS-R as a sample of choice, the optimization of the reaction conditions was carried out. First, the stirring rate was varied. It was found that the stirring in the range of 500–1300 rpm has no effect on the rate of BrAn transformation (see Figure S5). The conversion of BrAn about 92–94% was attained by 60 min of the reaction. All experiments were carried out at a stirring rate of 900 rpm.

The effect of catalyst loading in the range from 15 mg (0.14 mol.% of Pd) up to 50 mg (0.47 mol.% of Pd) was studied. It was found (Figure S6) that the initial BrAn transformation rate goes up proportionally to the increase of the catalyst loading, indicating an absence of diffusion disguises.

**Figure 7:**
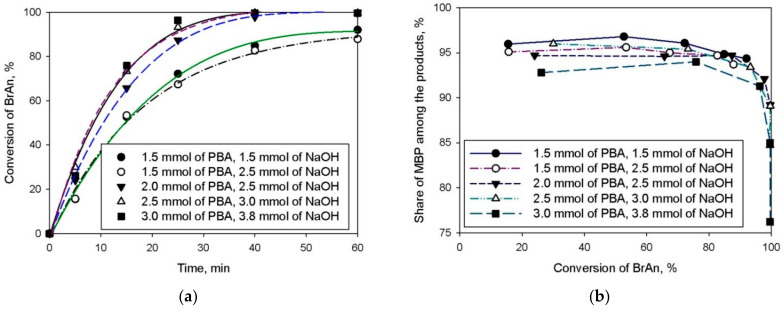
Influence of the amount of PBA and NaOH on the BrAn conversion vs. time (**a**) and on the “selectivity” to MBP vs. BrAn conversion (**b**); reaction conditions: 60 °C, 900 rpm, and 30 mg of 1%-Pd/HPS-R.

For the 1%-Pd/HPS-R catalyst a series of experiments was carried out at various amounts of PBA and NaOH but at a constant content of BrAn (1 mmol) at 60 °C and 900 rpm, using 30 mg (0.28 mol.% of Pd) of the catalyst. It was found (Figure 7a) that an increase in the amount of PBA from 1.5 mmol up to 2.5 mmol while maintaining 1.20–1.25-fold excess of NaOH with respect to PBA led to a gradual increase in BrAn conversion.

Complete conversion of BrAn was achieved by 40 min at the PBA content of 2.5 mmol and 3.0 mmol of NaOH. Further increase in the PBA content up to 3.0 mmol did not significantly influence the rate of BrAn transformation. As was indicated in Section 2.3, PBA is able to undergo homo-coupling, yielding BP as a product. This can result in the limitation of the cross-coupling reaction by PBA amount, especially at a high BrAn conversion. NaOH should also be used in excess, since it is required for PBA activation and neutralization of inorganic side-products of the reaction [34]. It is noteworthy that the increase in PBA amount results in a slight decrease in the overall process “selectivity” with respect to MBP, which is presented (Figure 7b) as a share of MBP among the organic reaction products (MBP and BP). In most of the experiments, the share of MBP was about 94% at 90% of BrAn conversion.

**Figure 8:**
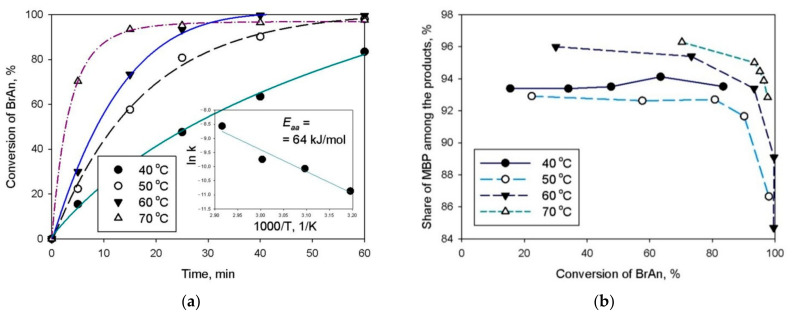
Influence of temperature on the dependence of BrAn conversion vs. time (**a**) and on the “selectivity” with respect to MBP vs. BrAn conversion (**b**); other reaction conditions: 1 mmol of BrAn, 2.5 mmol of PBA, 3.0 mmol of NaOH, 900 rpm, and 30 mg of reduced 1%-Pd/HPS-R.

The influence of temperature in the range of 40–70 °C on the BrAn conversion over 1%-Pd/HPS-R allowed estimating an apparent activation energy (*E_aa_*), which was found to be 64 kJ/mol (Figure 8a). This value is in good agreement with the reported data for Pd-catalyzed Suzuki cross-coupling [35,36]. It was also found that the increase in temperature leads to a concomitant increase in “selectivity” with respect to MBP, from 85% to about 92% at the conversion of BrAn close to 100% (see Figure 8b).

**Figure 9:**
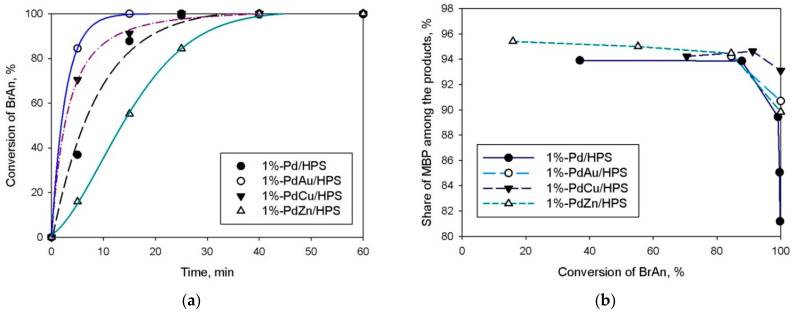

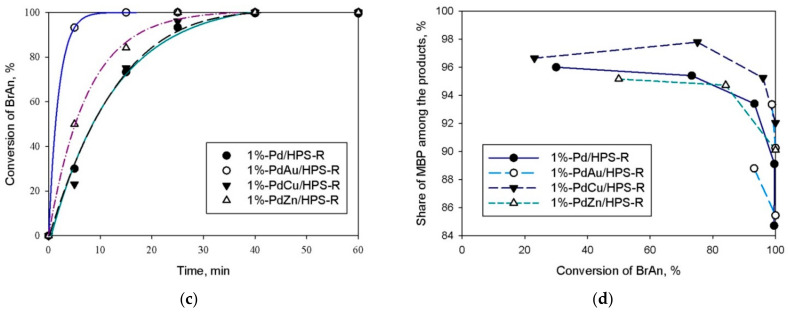
Dependence of BrAn conversion on time (**a**,**c**) and “selectivity” with respect to MBP vs. BrAn conversion (**b**,**d**) for synthesized mono- and bimetallic samples (in the catalyst’s designations “R” refers to preliminarily reduced samples); reaction conditions: 1 mmol of BrAn, 2.5 mmol of PBA, 3.0 mmol of NaOH, 60 °C, 900 rpm, and 30 mg of catalyst.

Optimization of the main reaction parameters allowed to choose the following reaction conditions for testing the synthesized bimetallic samples: 1 mmol of BrAn, 2.5 mmol of PBA, 3.0 mmol of NaOH, 60 °C, 900 rpm, and 30 mg of the catalyst; the results are summarized in Figure 9 and Table 2. It was shown that in the case of the initial (unreduced) catalysts, the highest activity was found for 1%-PdAu/HPS, while the lowest reaction rate was observed in the case of 1%-PdZn/HPS (Figure 9a,b). It is known that palladium and zinc acetates can form heterometallic complexes [37], which can likely complicate the formation of active Pd species in the Suzuki reaction. Unreduced 1%-PdCu/HPS showed moderate results.

Preliminarily reduction was found to strongly influence the catalytic properties of all the samples (Figure 9c, Table 2). After reduction (1%-PdCu/HPS-R), the activity of the copper-containing sample decreased more than two times. In general, lower activity of the nanoparticulate catalysts in comparison with the samples containing Pd^2+^ species is typical for the Suzuki reaction [38] and is due to the in situ transfer of Pd from NPs to homogeneous active forms. However, for the two other bimetallic samples (1%-PdAu/HPS-R and 1%-PdZn/HPS-R), the increase in catalytic activity was found after the reduction in hydrogen flow. In case of reduced 1%-PdZn/HPS-R, the formation of mixed Pd/Zn/ZnO NPs (Figure 6) likely resulted in an increase of the number active palladium sites (the effect of dilution of one metal with the other) or in the shift of adsorption energy of the reactant (BrAn) on palladium (electronic effect of zinc). The latter is a subject of DFT calculation, which is on-going and will be reported elsewhere.

The remarkable high activity of the preliminarily reduced 1%-PdAu/HPS-R and 0.5%-PdAu/HPS-R as compared to other catalysts can be explained by partial deposition of Pd on the surface of the preliminarily formed Au NPs and formation of core-shell PdAu NPs. Moreover, for these catalysts the amounts of PBA and NaOH can be decreased to 1.5 mmol without any detriment in activity (in contrast to 1%-Pd/HPS-R), which makes PdAu samples more beneficial from an environmental point of view.

**Figure 10:**
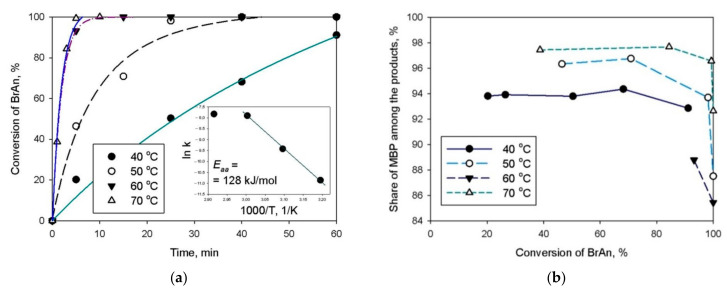
Influence of temperature on the dependence of BrAn conversion vs. time (**a**) and on the “selectivity” with respect to MBP (**b**): 1 mmol of BrAn, 2.5 mmol of PBA, 3.0 mmol of NaOH, 900 rpm, and 30 mg of reduced 1%-PdAu/HPS-R.

The effect of temperature was studied for the 1%-PdAu/HPS-R catalyst (Figure 10a) and the *E_aa_* value calculated in the range of 40–60 °C was 128 kJ/mol. This is double the value of the one found for the monometallic sample 1%-Pd/HPS-R (Figure 8a). While assuming the same reaction mechanism, a relatively high *E_aa_* along with a high observed reaction rate can be due to the increase in the number of active sites (increased pre-exponential factor in Arrhenius equation). It is noteworthy that the obtained value of *E_aa_* is in good agreement with the data reported by other research groups; e.g., Crawford et al. found high values of the apparent activation energy (111–116 kJ/mol) for the Suzuki reaction carried out using a carbene Pd^2+^ complex [39]. The temperature rise from 60 °C up to 70 °C did not give a measurable increase in the reaction rate, which may indicate an influence of diffusion. It is important to underline that the “selectivity” with respect to MBP (Figure 10b) increased with temperature.

**Figure 11:**
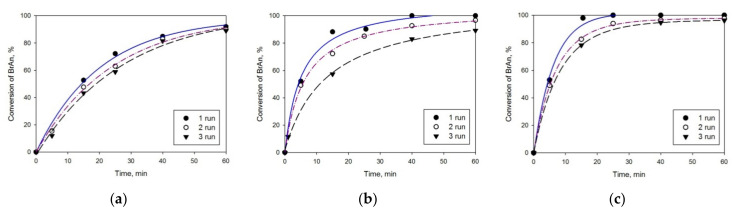
Influence of repeated runs on the dependence of BrAn conversion vs. time for 1%-Pd/HPS-R (**a**), 0.5%-PdAu/HPS-R (**b**), and 1%-PdAu/HPS-R (**c**): 1 mmol of BrAn, 1.5 mmol of PBA, 1.5 mmol of NaOH, 60 °C, 900 rpm, and 30 mg of catalyst.

Finally, the stability of the catalysts was addressed by their reuse in three repeated reaction runs (Figure 11). 1%-Pd/HPS-R catalyst was compared to 0.5%-PdAu/HPS-R and 1%-PdAu/HPS-R under the same reaction conditions: 1 mmol of BrAn, 1.5 mmol of PBA, 1.5 mmol of NaOH, 60 °C, 900 rpm, and 30 mg of catalyst.

It was found that all samples containing 1 wt.% of Pd (0.28 mol.% of Pd) revealed satisfactory stability at three consecutive runs: conversion of BrAn decreased from 92% to 89% in case of 1%-Pd/HPS-R and from 100% to 96% in case of 1%-PdAu/HPS-R. The 0.5%-PdAu/HPS-R was less stable: the conversion of BrAn decreased from 100% to 89% in three consecutive runs. Lower stability of 0.5%-PdAu/HPS-R can be due to the low metal loading (0.14 mol.% of Pd), which can result in faster deactivation for several reasons, including redistribution and aggregation of Pd NPs in the HPS pores, which was earlier observed for similar catalytic system [34], or because of slight metal leaching. For the catalyst 0.5%-PdAu/HPS-R, the hot-filtration test was carried out, which revealed no activity of the filtrate. This fact indicates that if even some Pd leaching occurred, the leached species were inactive in the cross-coupling process. In spite of the slight loss of activity of the PdAu samples, they can be considered as promising systems for Suzuki cross-coupling due to their higher reaction rates compared to the monometallic analogues.

## 4. Discussion

The results of our work reported herein demonstrate that HPS, which is chemically and mechanically stable, commercially available, and a relatively low-cost polymer, can serve as a medium for NPs formation. Both monometallic (Pd) and bimetallic (PdAu, PdCu and PdZn) catalysts containing 0.5–1.5 wt.% of Pd (data of XFA) and about 2 wt.% of a second metal were synthesized using HPS as a support. It was shown that independently in the presence of a second metal, palladium acetate is uniformly distributed in a highly porous aromatic network of HPS, which allows formation of small Pd NPs of about 2 nm in diameter after reduction in a hydrogen flow at 300 °C (the limit of temperature stability of the chosen HPS [40]). In the case of the Pd(CH_3_COO)_2_ precursor, such reduction conditions are suitable for fast growth of metallic NPs [41]. However, for other metal acetates used in this work, such as Cu(CH_3_COO)_2_ and Zn(CH_3_COO)_2_, reduction was not achieved, and the corresponding oxides were found on the surface of bimetallic catalysts. It is noteworthy that in contrast to copper acetate, the properties of zinc acetate are similar to palladium acetate in terms of its ability to form oligomers, increasing its hydrophobicity and allowing its uniform distribution inside a porous aromatic support (HPS). Thus, the reduction of bimetallic 1%-PdZn/HPS catalyst results in the formation of small (2.0 ± 0.5 nm) Pd/Zn/ZnO NPs. The presence of metallic zinc in the reduced 1%-PdZn/HPS-R sample was confirmed by the BE positive shift in the Pd^0^ HR-XPS spectra.

When HPS was preliminarily loaded with Au NPs of about 20 nm in diameter, the impregnation by Pd acetate with further reduction in hydrogen flow at 300 °C resulted in the formation of small Pd NPs (about 2 nm) and also core-shell PdAu NPs. We suppose that the palladium shell is extremely thin (2–3 atomic layers) and hardly detectable. The results of the higher catalytic activity of PdAu/HPS in the Suzuki reaction, as compared to monometallic catalysts, evidenced the synergy of Pd and Au, suggesting the formation of PdAu NPs.

All the synthesized mono- and bimetallic catalysts were tested in a model reaction of Suzuki cross-coupling between BrAn and PBA under relatively mild conditions using an EtOH-H_2_O mixture (4:1) as a solvent. PdAu/HPS catalysts containing about 0.5 wt.% and 1 wt.% of Pd were 3-fold more active as compared to 1%-Pd/HPS. Furthermore, the 1%-PdAu/HPS-R sample revealed a doubled *E_aa_* value, which likely indicated a change in the nature of the palladium active centers (note that Au is inert in Suzuki reaction) and can also serve as evidence of PdAu NPs formation. Moreover, PdAu catalysts allowed noticeably decreased amounts of PBA and NaOH in order to reach a close to 100% BrAn conversion as compared to the monometallic Pd sample. At the same time, the stability of the PdAu/HPS catalysts did not change and was comparable to a typical Pd/HPS catalyst.

The 1%-PdCu/HPS catalyst demonstrated higher activity as compared to 1%-Pd/HPS; however, after the reduction its activity strongly decreased. No interaction between the Cu-containing phase and Pd was detected. In contrast, the 1%-PdZn/HPS catalyst revealed higher activity even after being reduced, which was associated with Pd-Zn intimal interactions, resulting in the formation of PdZn alloy NPs. Thus, for the first time we have demonstrated that PdZn alloys are promising catalysts for Suzuki cross-coupling.

It is noteworthy that the most active PdAu/HPS samples overperformed in terms of the activity/selectivity of other reported catalysts. Many works describing the behavior of ligandless catalysts in the Suzuki cross-coupling reaction reported a palladium loading of 0.5–1 mol.% or higher, in order to reach a complete conversion of the aryl halides [9,14,42,43]. We herein demonstrated that HPS gives highly active catalysts at Pd concentrations of about 0.14–0.28 mol.%. At present, such a low Pd loading in the cross-coupling processes is of high importance and can be implemented using polymer-based catalysts [44,45]. The activity of the synthesized Pd/HPS catalysts is inferior to Pd supported on PAFs (porous aromatic frameworks) [4], but PAFs itself have a rather complex synthesis procedure.

Finally, we would like to underline that hyper-cross-linked polymers have a tunable structure, which may further increase the activity, selectivity, and stability in cross-coupling processes; this work is in progress and will be reported elsewhere.

## Figures and Tables

**Table 1:** SSA of the initial HPS and fresh mono- and bimetallic catalysts obtained by liquid nitrogen physisorption.

Sample	SSA_BET_, m^2^/g	SSA_t-plot_, m^2^/g ^1^
HPS	793	189 ^2^; 603 ^3^
1%-Pd/HPS	668	104 ^2^; 572 ^3^
1%-Pd/HPS-R	595	133 ^2^; 459 ^3^
0.5%-PdAu/HPS	713	141 ^2^; 593 ^3^
0.5%-PdAu/HPS-R	655	148 ^2^; 507 ^3^
1%-PdAu/HPS	744	187 ^2^; 556 ^3^
1%-PdCu/HPS	872	123 ^2^; 747 ^3^
1%-PdCu/HPS-R	414	264 ^2^; 148 ^3^
1%-PdZn/HPS	851	179 ^2^; 672 ^3^
1%-PdZn/HPS-R	732	166 ^2^; 588 ^3^

^1^ SSA_BET_ and SSA_t-plot_—specific surface areas calculated by BET and t-plot methods, respectively; ^2^ external SSA; ^3^ SSA of micropores.

**Table 2:** Catalytic results for the synthesized mono- and bimetallic samples (1 mmol of BrAn, 2.5 mmol of PBA, 3.0 mmol of NaOH, 60 °C, 900 rpm, and 30 mg of catalyst).

Sample	Maximum Conversion of BrAn, %	Yield of MBP, %	*R*_0_, mol_BrAn_/(mol_Pd_·min) ^2^
1%-Pd/HPS	99	89	26.2
1%-Pd/HPS-R	~100	89	21.3
1%-Pd/HPS-R ^1^	92	87	11.0
0.5%-PdAu/HPS-R	99	93	63.0
0.5%-PdAu/HPS-R ^1^	**100**	**92**	**61.6**
1%-PdAu/HPS	100	91	66.6
1%-PdAu/HPS-R	100	85	73.5
1%-PdAu/HPS-R ^1^	**~100**	**94**	**65.9**
1%-PdCu/HPS	100	93	38.5
1%-PdCu/HPS-R	100	92	13.7
1%-PdZn/HPS	100	89	8.7
1%-PdZn/HPS-R	100	90	23.6

^1^ 1.5 mmol of PBA, 1.5 mmol of NaOH, and other conditions are as indicated in the table heading; ^2^ *R*_0_ was calculated as a tangent of the initial slope divided to overall Pd content (see Section 2.3).

## Data Availability

Data sharing is not applicable to this article.

## Supplementary Materials

The following supporting information can be downloaded at https://www.mdpi.com/article/10.3390/nano12010094/s1, Figure S1: Adsorption-desorption isotherms (a,b) and pore volume distribution (c,d) for the initial HPS (black cyrcles) and catalyst samples: unreduced 1%-PdAu/HPS and reduced 1%-PdAu/HPS-R (red circles); 1%-PdCu/HPS and 1%-PdCu/HPS-R (green triangles); 1%-PdZn/HPS and 1%-PdZn/HPS-R (yellow triangles), Figure S2: EDX data for 0.5%-PdAu/HPS-R sample in area 1 (a) and area 2 (b) of corresponding HAADF STEM image, Figure S3: High-resolution XPS spectra of Au 4f (a), Cu 2p (c), Zn 2p (e) and Pd 3d (b,d,f) in the initial (unreduced) samples: 1%-PdAu/HPS(a,b), 1%-PdCu/HPS(c,d) and 1%-PdZn/HPS(e,f), Figure S4: High-resolution XPS spectra of Au 4f (a), Cu 2p (c), Zn 2p (e) and Pd 3d (b,d,f) in the reduced samples: 1%-PdAu/HPS-R (a,b), 1%-PdCu/HPS-R(c,d) and 1%-PdZn/HPS-R (e,f), Figure S5: Influence of stirring rate on the dependence of BrAn conversion vs. time for 1%-Pd/HPS-R (catalyst loading 30 mg, 1 mmol of BrAn, 1.5 mmol of PBA, 1.5 mmol of NaOH, 60 °C), Figure S6: Influence of catalyst 1%-Pd/HPS-R loading on the dependence of BrAn conversion vs. time (1 mmol of BrAn, 1.5 mmol of PBA, 1.5 mmol of NaOH, 60 °C), Method of the reaction rate calculation.
