# Supplementary material for: Mono- and Bimetallic Nanoparticles Stabilized by an Aromatic Polymeric Network for a Suzuki Cross-Coupling Reaction
Source: Nanomaterials (Basel). 2021 Dec 29;12(1):94. doi: 10.3390/nano12010094 (PMC8746394; doi:10.3390/nano12010094)
Supplement: Supplementary file 1 [file nanomaterials-12-00094-s001.zip › nanomaterials-1466646-supplementary.pdf]

nanomaterials

MDPI

Supplementary Materials

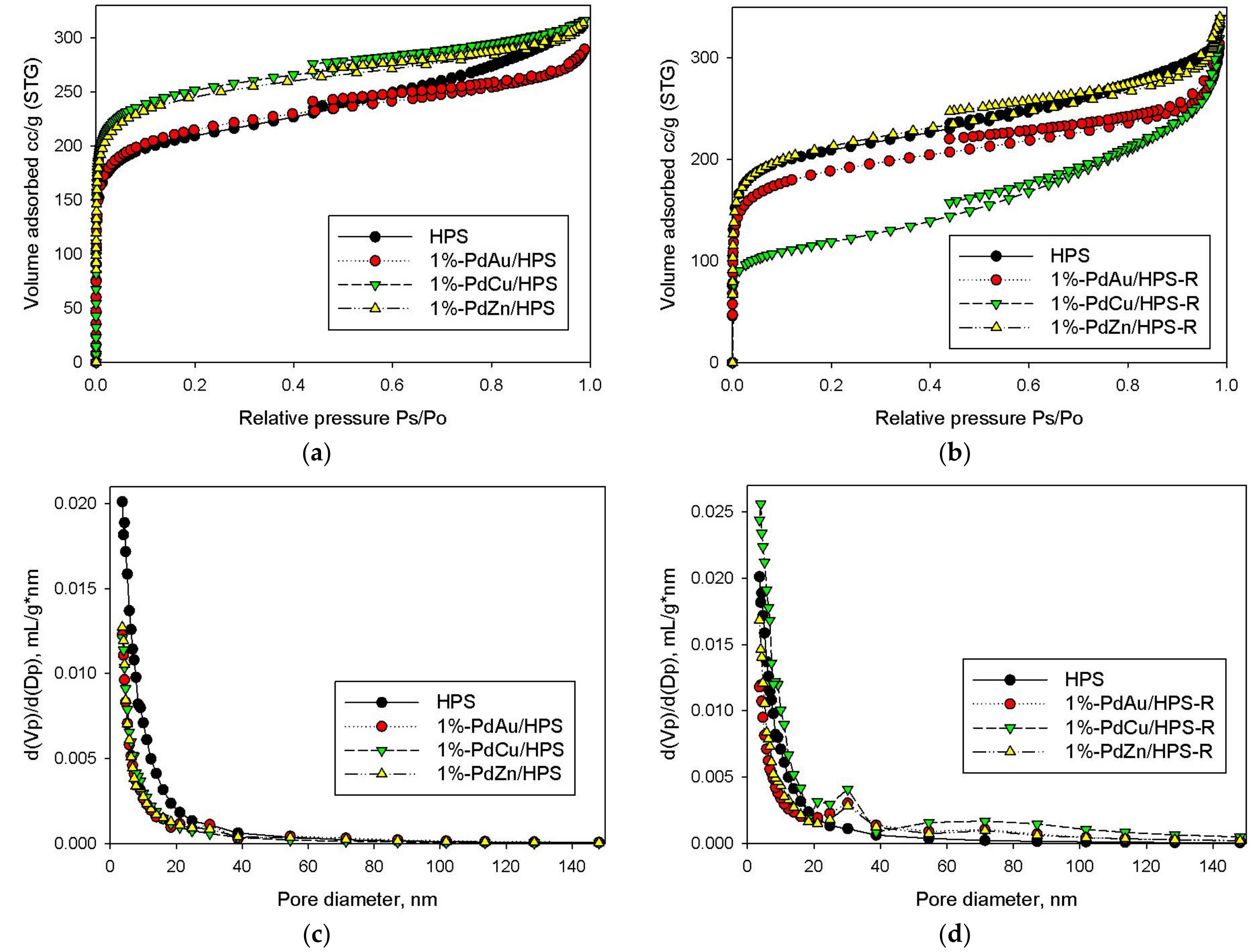

Figure S1. Adsorption-desorption isotherms (**a, b**) and pore volume distribution (**c, d**) for the initial HPS (black cyrcles) and catalyst samples: unreduced 1%-PdAu/HPS and reduced 1%-PdAu/HPS-R (red circles); 1%-PdCu/HPS and 1%-PdCu/HPS-R (green triangles); 1%-PdZn/HPS and 1%-PdZn/HPS-R (yellow triangles).

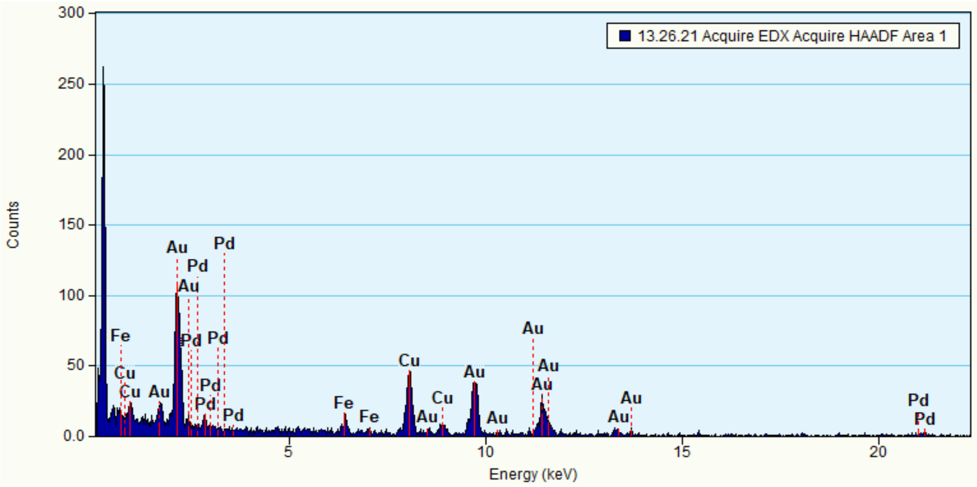

(**a**)

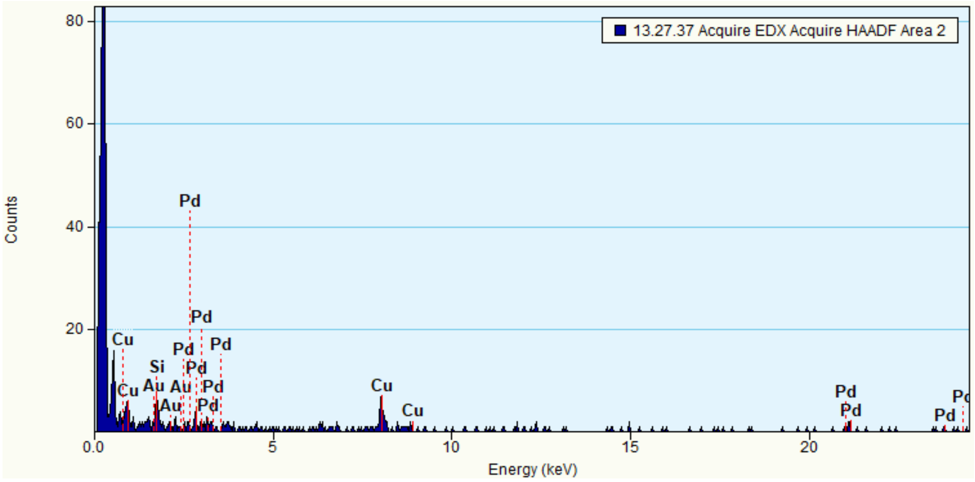

(**b**)

Figure S2. EDX data for 0.5%PdAu/HPS-R sample in area 1 (**a**) and area 2 (**b**) of corresponding HAADF STEM image.

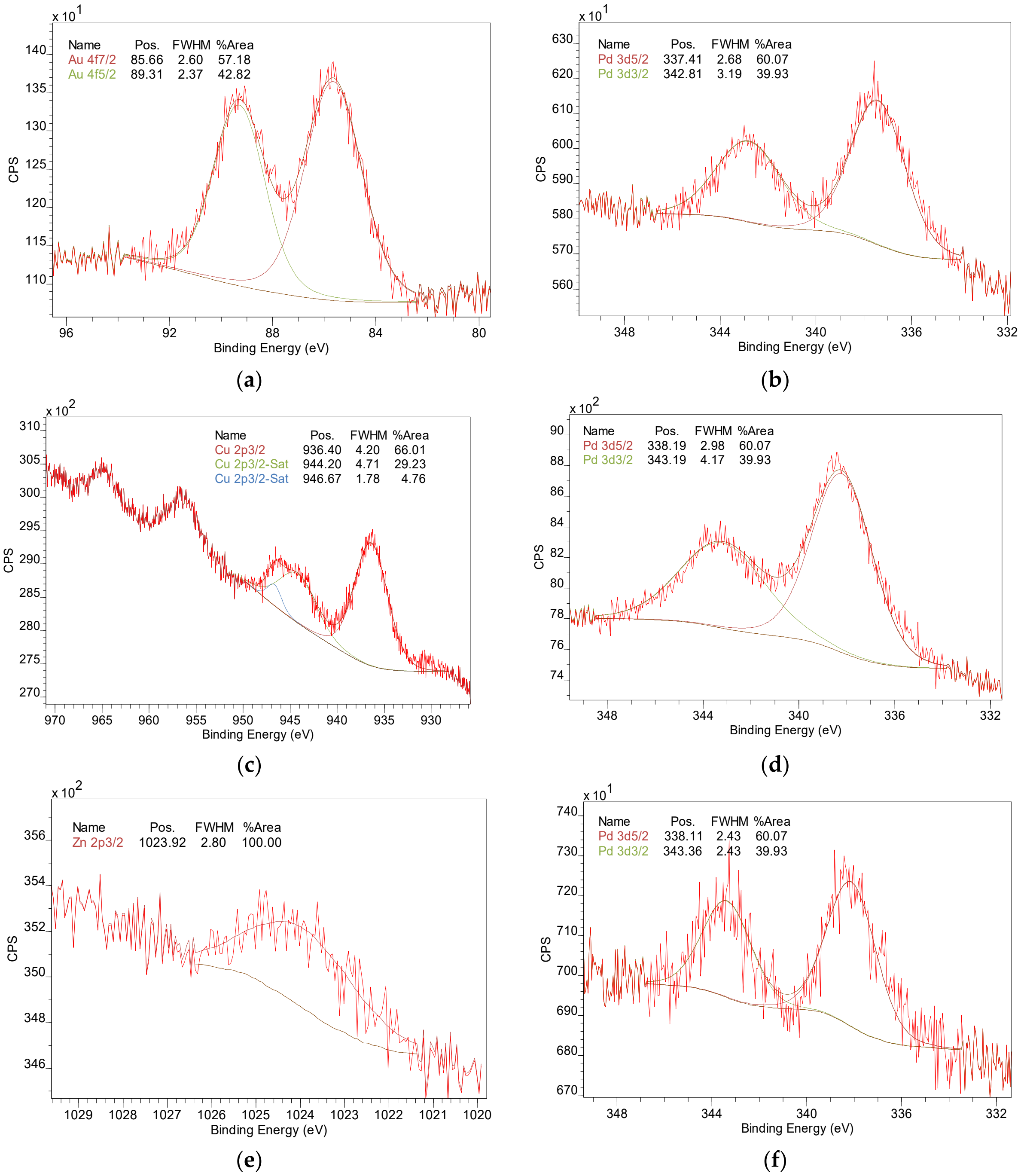

Figure S3. High-resolution XPS spectra of Au 4f (**a**), Cu 2p (**c**), Zn 2p (**e**) and Pd 3d (**b, d, f**) in the initial (unreduced) samples: 1%-PdAu/HPS (**a, b**), 1%-PdCu/HPS (**c, d**) and 1%-PdZn/HPS (**e, f**).

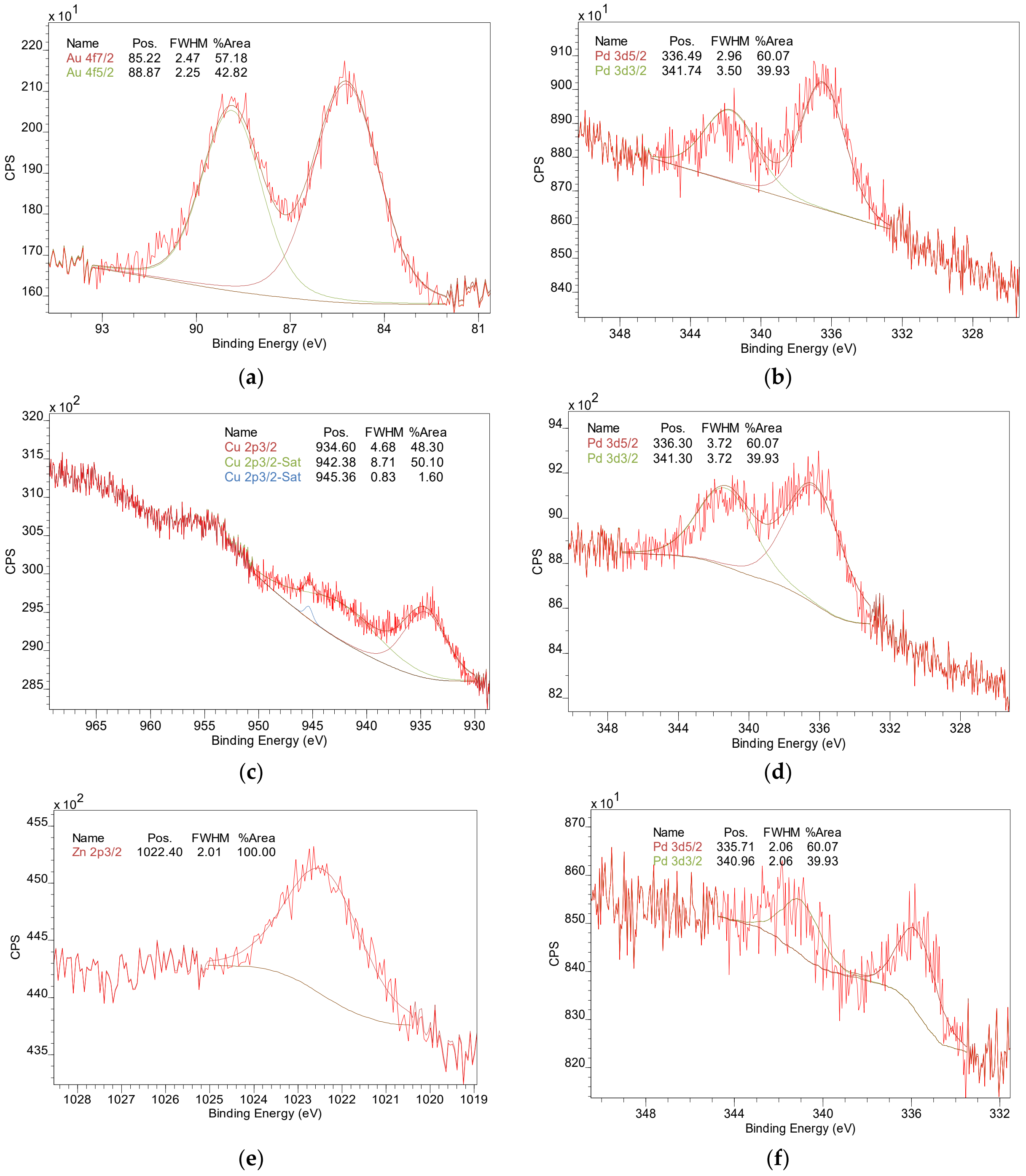

Figure S4. High-resolution XPS spectra of Au 4f (**a**), Cu 2p (**c**), Zn 2p (**e**) and Pd 3d (**b, d, f**) in the reduced samples: 1%-PdAu/HPS-R (**a, b**), 1%-PdCu/HPS-R (**c, d**) and 1%-PdZn/HPS-R (**e, f**).

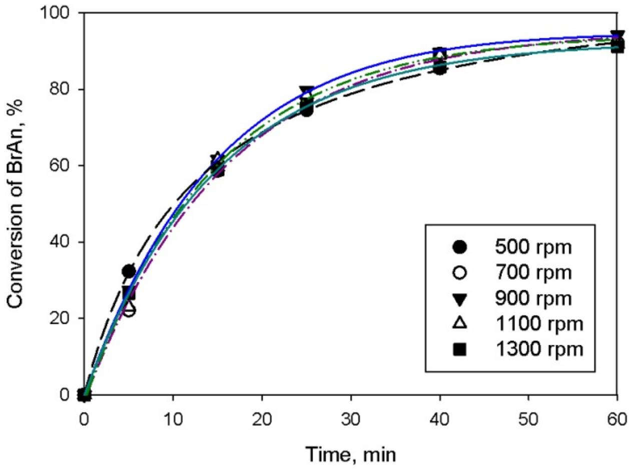

Figure S5. Influence of stirring rate on the dependence of BrAn conversion *vs.* time for 1%-Pd/HPS-R (catalyst loading 30 mg (0.28 mol.%), 1 mmol of BrAn, 1.5 mmol of PBA, 1.5 mmol of NaOH, 60°C).

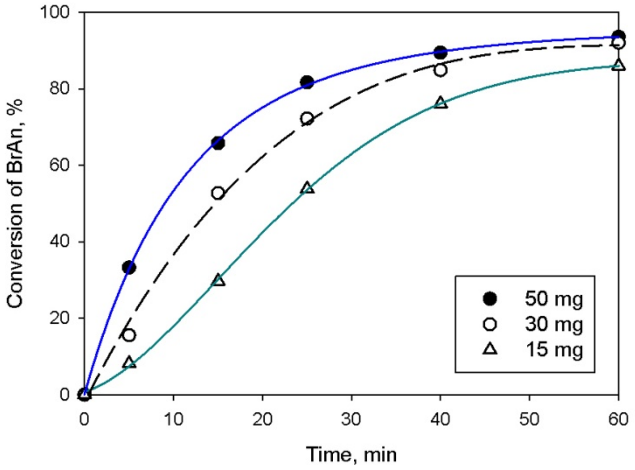

Figure S6. Influence of catalyst 1%-Pd/HPS-R loading on the dependence of BrAn conversion *vs.* time (1 mmol of BrAn, 1.5 mmol of PBA, 1.5 mmol of NaOH, 60°C, 900 rpm).

Method of the reaction rate of calculation.

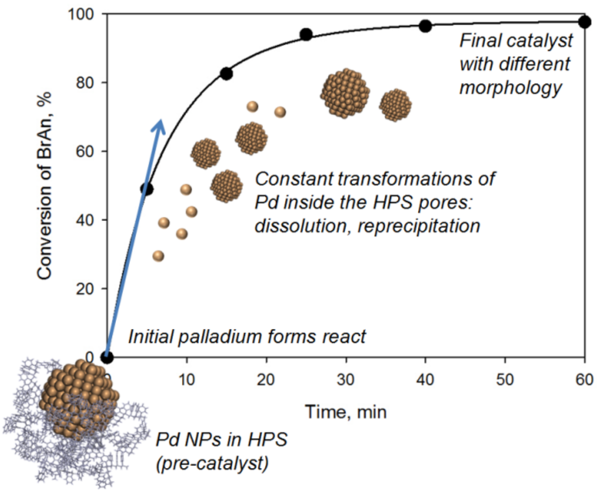

Catalytic activity was characterized by the initial reaction rate (*R_0*), which is a tangent of the slope of the initial linear part on kinetic curves of BrAn consumption related to Pd amount in the reaction mixture.

Any form of palladium (Pd^{2+} or Pd^0) can be in the catalyst composition. However, Pd fastly react to form active species in situ, which further undergo precipitation-dissolution-reprecipitation (either on the support or on the existing nanoparticles).

Since catalysts constantly changing their morphology during catalysis, it is important to compare the initial parts of kinetic curves of aryl halides conversion.
